# ClC-1 chloride channels: state-of-the-art research and future challenges

**DOI:** 10.3389/fncel.2015.00156

**Published:** 2015-04-27

**Authors:** Paola Imbrici, Concetta Altamura, Mauro Pessia, Renato Mantegazza, Jean-François Desaphy, Diana Conte Camerino

**Affiliations:** ^1^Department of Pharmacy - Drug Sciences, University of Bari “Aldo Moro”,Bari, Italy; ^2^Department of Experimental Medicine, School of Medicine, University of Perugia,Perugia, Italy; ^3^Neuroimmunology and Neuromuscular Diseases Unit, IRCCS Fondazione Istituto Neurologico “Carlo Besta”,Milano, Italy

**Keywords:** ClC-1 chloride channel, skeletal muscle physiology, myotonia congenita, ion channel pharmacology, skeletal muscle plasticity

## Abstract

The voltage-dependent ClC-1 chloride channel belongs to the CLC channel/transporter family. It is a homodimer comprising two individual pores which can operate independently or simultaneously according to two gating modes, the fast and the slow gate of the channel. ClC-1 is preferentially expressed in the skeletal muscle fibers where the presence of an efficient Cl^-^ homeostasis is crucial for the correct membrane repolarization and propagation of action potential. As a consequence, mutations in the *CLCN1* gene cause dominant and recessive forms of myotonia congenita (MC), a rare skeletal muscle channelopathy caused by abnormal membrane excitation, and clinically characterized by muscle stiffness and various degrees of transitory weakness. Elucidation of the mechanistic link between the genetic defects and the disease pathogenesis is still incomplete and, at this time, there is no specific treatment for MC. Still controversial is the subcellular localization pattern of ClC-1 channels in skeletal muscle as well as its modulation by some intracellular factors. The expression of ClC-1 in other tissues such as in brain and heart and the possible assembly of ClC-1/ClC-2 heterodimers further expand the physiological properties of ClC-1 and its involvement in diseases. A recent de novo *CLCN1* truncation mutation in a patient with generalized epilepsy indeed postulates an unexpected role of this channel in the control of neuronal network excitability. This review summarizes the most relevant and state-of-the-art research on ClC-1 chloride channels physiology and associated diseases.

## Introduction

CLC proteins are ubiquitous chloride channels/transporters playing important roles in many physiological and physiopathological processes (Hille, 1992). Their story began with the electric fish, which expresses a high number of voltage-gated chloride channels in the cell of an electric organ originated from the skeletal muscle tissue (White and Miller, 1979). Due to the lack of specific inhibitors and the difficulties of biochemical purification, it was only in the 1990s that a molecular identification of voltage-gated chloride channels was made. In these years, the chloride channel from *Torpedo marmorata*, called ClC-0, was cloned with an elegant but extremely sophisticated expression cloning strategy (Jentsch et al., 1990). This allowed the description of a large family of CLC proteins homologous to ClC-0 and expressed in organisms within the kingdoms of life from eubacteria to animals (Jentsch et al., 1999; Maduke et al., 2000). These proteins exhibit a high degree of evolutionary conservation and function as homodimers (Middleton et al., 1996). In mammalian tissues, nine different CLC proteins have been identified, divided into plasma membrane Cl^-^ channels and vesicular Cl^-^/H^+^-exchangers (Jentsch, 2015).

The ClC-1 channel was the first member of the CLC family cloned in mammals (Steinmeyer et al., 1991a). In humans, the *CLCN1* gene is located on chromosome 7q35 and codes for a protein of ∼990 amino acids in length. This channel is mainly expressed in the skeletal muscle, where it supports the large chloride conductance of sarcolemma. Its physiological role was discovered by analyzing a mouse model of myotonia congenita (MC), a genetic disease of the skeletal muscle characterized by a reduced sarcolemma chloride conductance. A plethora of mutations in the *CLCN1* gene are known to produce dominant and recessive myotonia in humans and other animals. At lower levels it has been detected in kidney, heart, smooth muscle, and, more recently, in the central nervous system (Steinmeyer et al., 1991a; Chen et al., 2013). This review describes our current knowledge regarding ClC-1 channels, as a example for using human genetic diseases and mouse models to facilitate the elucidation of the cellular roles of ion channels, and the research challenges these proteins continue to offer twenty-five years after the cloning of the first CLC.

## Molecular Structure and Function of ClC-1

**FIGURE 1:**
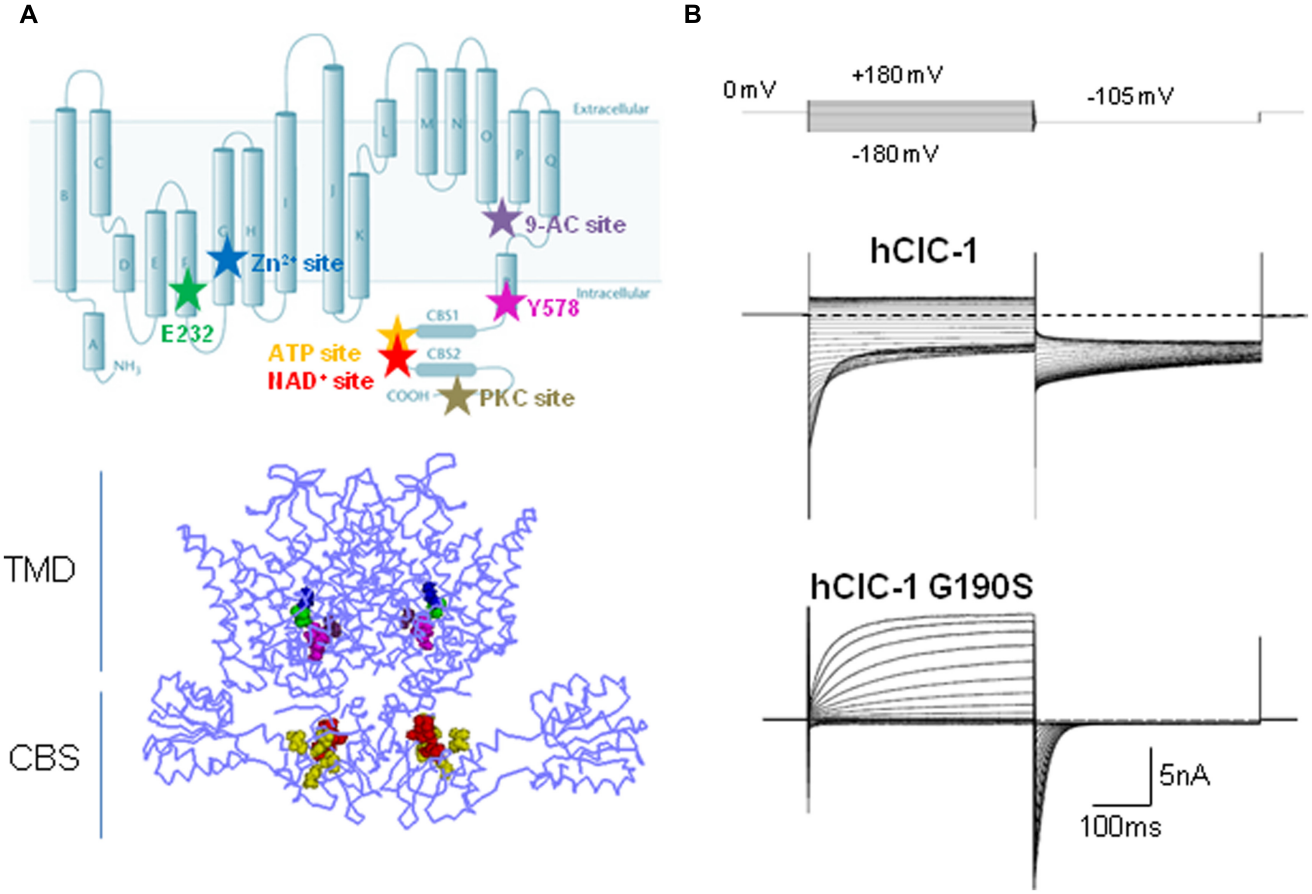
**Structure, function, and modulation of hClC-1 channels**. **(A)** Upper panel, diagram showing residues relevant for ClC-1 channel gating, E232 (green) and Y578 (magenta), and putative binding sites for Zn^2+^ (C277, blue), 9-AC (S537, violet), ATP (V613, V634, V860, E865, yellow), NAD^+^ (T636, H847, L848, red), PKC (Thr891, Ser892, Thr893, gray). Lower panel, three dimensional front view of hClC-1 channel modeled upon the structure of CmClC, including relevant residues for channel function and regulation. **(B)** Representative chloride currents recorded from tsA cells transfected with cDNAs coding for hClC-1 WT (up) and for a MC mutant hClC-1 G190S (down), in the whole-cell configuration of patch clamp. Note that G190S shows a strong outward rectification differently from WT. Cells were held at 0 mV and 400 ms voltage pulses were applied from -180 to +180 mV in 10-mV intervals every 3 s, in high chloride intracellular solution.

The ClC-1 voltage-gated chloride channel belongs to the CLC family, which comprises nine members in humans, named ClC-1 through ClC-7, plus ClC-Ka and ClC-Kb. They were initially assumed to behave as chloride ion channels, but successive experiments revealed that five out of the nine hClCs (ClC-3 through ClC-7) rather function as anion-proton exchangers (Picollo and Pusch, 2005; Scheel et al., 2005; Neagoe et al., 2010; Leisle et al., 2011; Guzman et al., 2013). Experimental data also support a close structural similarity among the members of the CLC family. Actually, most of the current information about the 3D structure of ClC-1 channels have been derived from the analysis of CLC anion-proton exchangers crystallized from several prokaryotic (EcCLC) and eukaryotic species (CmCLC; **Figure 1A**; Dutzler et al., 2002; Accardi et al., 2006; Lobet and Dutzler, 2006; Feng et al., 2010; Robertson et al., 2010; Jayaram et al., 2011; Lim et al., 2012). The ClC-1 channel is a dimer of two homologous subunits, each forming a chloride ion conducting pore independent from the other, allowing ClC-1 to function as a “double-barrel” channel (Saviane et al., 1999). Each subunit consists of 18 domains (helices A-R) and two tandem cystathionine-β-synthase (CBS) domains located in the intracellular C-terminal (Meyer and Dutzler, 2006; Markovic and Dutzler, 2007). According to the 3D structures, membrane helices D, F, N, and R contribute to the ClC ion transport pathway, helices H, I, P, and Q form part of the interface between the two monomers, and helix R connects the transmembrane segments of the channel to the C-terminal domain (Dutzler et al., 2002, 2003; Feng et al., 2010). Each pore contains three separate chloride ion binding sites, named S_int_, S_cen_, and S_ext_ according to their position relative to the extra- or intracellular side of the plasma membrane. The S_cen_ position forms a significant part of the selectivity filter where conserved and less well conserved aminoacids coordinate the chloride ion within the conduction pathway (Dutzler et al., 2002, 2003; Lobet and Dutzler, 2006).

Early single channel recordings of ClC-1 channels expressed in heterologous systems, demonstrate two conductance states and confirmed the existence of two ion conduction pathways for homodimeric ClC channel, as shown for ClC-0 (Miller, 1982; Miller and White, 1984; Pusch et al., 1994; Saviane et al., 1999). Whole cell recordings of hClC-1 channels in high chloride intracellular solution show chloride currents that rapidly activate and deactivate at negative potentials and that saturate at positive potentials (**Figure 1B**).

Channel opening in ClC-1 is regulated by two distinct gating patterns: fast (or single-pore) and slow (or common-pore) gating (Accardi and Pusch, 2000; Dutzler, 2006), fast gating being about 10-fold faster than common gating (Miller, 1982; Saviane et al., 1999; Stölting et al., 2014b). The slow gate controls the opening and closing of the two subunits simultaneously, whereas the fast gate regulates the function of independent pores (Saviane et al., 1999). The carboxyl side chain of glutamate residue E232 of helix F is the main responsible for the fast gating: it projects into the extracellular-facing portion of the ion conduction pathway and functions as the voltage-, Cl^-^- and H^+^-activated fast gate, disclosing S_ext_ when protonated (Zifarelli and Pusch, 2007). This residue is part of the Gly-Lys/Arg-Glu-Gly-Pro sequence motif that is conserved in all CLC channels apart from the kidney channels ClC-Ka and CIC-Kb. The molecular identity of the slow gate has still to be elucidated, and how the slow gate closes simultaneously both pores is still unknown. Far from simply involving helices and residues at the dimer interface, the emerging view on the mechanism of cooperative gating predicts the occurrence of concerted conformational changes involving the residue E232 in the channel pore, the intra-membrane interface between two monomers, and the C-terminal CBS domains via helix R (Duffield et al., 2003; Estévez et al., 2004; Fialho et al., 2007; Cederholm et al., 2010; Feng et al., 2010; Ma et al., 2011; Bennetts and Parker, 2013). Recently, it has been proposed that the slow channel closure depends on the H-bond formation between the E232 of pore helix F and the Y578 of the linker helix R (Bennetts and Parker, 2013). This H-bond interaction would enable the occlusion of the central anion binding site of the channel, thus hindering Cl^-^ flow.

The intracellular C-terminal of ClC-1 is a poorly characterized region. This portion, comprising two tandem CBS domains, is common to the eukaryotic CLC members, and appears to be relevant for ClC-1 expression, slow gating and intracellular modulation (**Figure 1A**; Meyer and Dutzler, 2006; Markovic and Dutzler, 2007; Tseng et al., 2007; Zhang et al., 2008; Hsiao et al., 2010; Bennetts et al., 2012; Bennetts and Parker, 2013). The crystal structure of the eukaryotic CLC protein demonstrates that CBS2 lies functionally close to the intramembrane domains while CBS1 faces into the cytoplasm (Feng et al., 2010). CBS2 interacts extensively with the R-helix linker and helix D, and with an intracellular loop linking helices H and I. By virtue of its direct connection to the C-terminus, the R helix could provide a pathway by which intracellular domain conformational changes regulate CLC activity during cell signaling events or modulate slow gating (Dutzler et al., 2002; Bykova et al., 2006; Garcia-Olivares et al., 2008; Feng et al., 2010; Ma et al., 2011; Stölting et al., 2014b). The presumed regulatory role of the CBS linker, with its highly variable and largely unstructured sequence, has not been assessed jet.

To date, no report exists showing that ClC-1 can form heteromers with members of the same family *in vivo*. However, as ClC-2 is ubiquitously expressed, the spontaneous formation of heterodimeric ClC-1-ClC-2 channels would be not surprising in native human tissues, where both channels co-exist under physiological conditions. ClC-1 and ClC-2 chloride channels differ profoundly in the voltage dependence of fast and slow gating. Whereas fast and slow opening of ClC-1 is stimulated by membrane depolarization (Stölting et al., 2014a), fast and cooperative gates of ClC-2 are closed at depolarized potentials and open upon membrane hyperpolarization (Stölting et al., 2014a). Therefore, an attempt to clarify the mechanism of cooperative interaction between subunits has been the functional characterization of “artificial” heterodimeric ClC-1-ClC-2 channels consisting of two functionally distinct ClC pores (Lorenz et al., 1996; Stölting et al., 2014a). The observation that ClC-1-ClC-2 heterodimeric channels lack cooperative gating (Stölting et al., 2014a) supports the idea that slow gating of CLC channels ultimately arises from conformational changes within an individual pore (Cederholm et al., 2010; Bennetts and Parker, 2013; Stölting et al., 2014b). Therefore, fast and slow gating are now supposed to be closely coupled and not independent processes, as previously thought.

### Modulatory Pathways

The structural bases of ClC-1 gating have been extensively studied as well as the contribution of ClC-1 channels to skeletal muscle physiology. How ClC-1 proteins are modulated by cell signaling pathways is, however, less well understood. There are compelling evidence that ClC-1 channel is finely regulated by the metabolic state of skeletal muscle fibers under physiological conditions, and its physiological contribution to skeletal muscle excitability must be read in the context of intracellular adenosine triphosphate (ATP) content, changes of the cytosolic redox state, alteration of the β-nicotinamide adenine dinucleotide (NAD^+^/NADH) ratio, membrane depolarization, acidification in exercised muscle, and protein kinases modulation.

The direct effect of cytoplasmic ATP on ClC-1 channels has been studied by several laboratories with apparently controversial results (Bennetts et al., 2007; Tseng et al., 2007; Zifarelli and Pusch, 2008). Indeed, Tseng et al. (2007) and Bennetts et al. (2007) provided evidence that ATP directly inhibits recombinant ClC-1 channels by shifting the voltage dependence of slow gating to positive potentials, an effect that is enhanced by low intracellular pH (pH_i_). This experimental finding provided a reasonable explanation for previously published effects of pH_i_ on chloride currents in different biological systems (Saviane et al., 1999; Pedersen et al., 2004, 2005). Indeed, reducing pH_i_ was shown to slow deactivation and increase the open probability of heterologously expressed ClC-1 channels (Rychkov et al., 1996; Saviane et al., 1999; Accardi and Pusch, 2000). Conversely, the acidification of skeletal muscle cells was reported to decrease sarcolemmal chloride conductance, thus maintaining the essential excitability in the partially depolarized sarcolemma and limiting muscle fatigue (Pedersen et al., 2004, 2005). ATP binding to the intracellular side of ClC-1 C-terminus could be the prerequisite required to observe a reduction of the ClC-1 conductance at low pH_i_ in intact muscle fibers, thus reconciling these apparently divergent results (Bennetts et al., 2007; Tseng et al., 2007). Despite these exhaustive experiments, Zifarelli and Push questioned the direct pH_i_-dependent ATP modulation of ClC-1, as they failed to detect any ATP block in inside-out patches from *Xenopus* oocytes expressing ClC-1 channels (Zifarelli and Pusch, 2008). The hidden culprit underlying this further controversy seems to rely on the oxidation of ClC-1. Indeed, it seems that the ATP-mediated inhibition of ClC-1 channels is dependent on the redox-state of the cell, disappearing in the oxidizing condition of excised inside-out patches whereas being evident when adding reducing agents to the patch (Zhang et al., 2008). Oxidation of ClC-1 might thus be one of the mechanisms that control the muscle response to fatigue, by regulating the ATP inhibition of ClC-1 (Zhang et al., 2008). As ATP, also NAD^+^ has been shown to affect the slow channel gating in a pH_i_-dependent mode by binding to the CBS domains of ClC-1 (Bennetts et al., 2012). Through mutagenesis experiments, relevant residues within both CBS domains forming a putative binding site for both ATP and NAD^+^ have been highlighted (**Figure 1A**; Bennetts et al., 2007; Tseng et al., 2007). Zn^2+^ ions are also known to block ClC-1 channels by facilitating closing of the slow gate of ClC-1 (Duffield et al., 2005). This effect is eliminated by the mutation C277S which greatly increases the minimal open probability of the slow gate in ClC-1 channels, and is facilitated by the mutant V321A, which reduces the open probability of slow gating (Accardi et al., 2001; Duffield et al., 2005).

Several pharmacological and molecular studies indicate that ClC-1 is regulated by protein phosphorylation events (Brinkmeier and Jockusch, 1987; Bryant and Conte-Camerino, 1991; Tricarico et al., 1991; Rosenbohm et al., 1999; Papponen et al., 2005; Camerino et al., 2014). The external application of 4β-phorbol esters on HEK 293 cells transfected with hClC-1 channels reduced the instantaneous whole-cell current amplitude (Rosenbohm et al., 1999). This effect was abolished when the cells were intracellularly perfused with a specific protein kinase C (PKC) inhibitor, chelerythrine, suggesting that the effect of 4β-phorbol esters was mediated by PKC. The C-terminal region seems at least in part responsible for the effect of PKC activation on ClC-1 channels: indeed, several serine residues have been identified in the C-terminal domain which mediate PKC-dependent inhibition of ClC-1 chloride currents (**Figure 1A**; Hsiao et al., 2010). Consistently, the chloride conductance of sarcolemma at rest (gCl), which is mainly supported by ClC-1, was shown to be reduced by activation of PKC or increased by inhibitors of PKC. Such an effect may have important implications for skeletal muscle function (Bryant and Conte-Camerino, 1991; De Luca et al., 1998; Pierno et al., 2007; Pedersen et al., 2009b; Camerino et al., 2014). Phosphorylation–dephosphorylation signaling was shown to be relevant also for the correct targeting of ClC-1 to sarcolemma in skeletal muscle fibers (Papponen et al., 2005). Furthermore, electrophysiological studies showed an increase of gCl in the PKC𝜃-null mice with respect to wild-type, and a consequent reduction of muscle excitability (Camerino et al., 2014). Regulation of ClC-1 channels through PKC-dependent phosphorylation appeared to be involved in the muscular effects of a number of growth factors, hormones, and drugs, including the growth hormone, insulin-like growth factor-1 (IGF-1), ghrelin, angiotensin II, growth hormone secretagogues, and statins (De Luca et al., 1994b, 1998; Pierno et al., 2003; Cozzoli et al., 2014).

The hypothesis that accessory subunits may exist and bind to the C-terminal region, as shown for other channels, cannot be excluded as well as the possible interaction of the carboxyl tail of ClC-1 with cytoskeletal elements in muscle cells. Such interactions may be necessary for the integrity of the cytoskeleton as well as for the homeostatic control of sarcolemmal ClC-1 content or function.

## Physiological Roles of ClC-1

### Muscle Excitability

The exact localization of ClC-1 channels in the skeletal muscle membrane is still debated (Lueck et al., 2010; Lamb et al., 2011). Studies performed on skinned muscle fibers provided evidence that the majority of the chloride conductance is located in the T-system (Dutka et al., 2008). This result is not surprising if one considers that a large Cl^-^ conductance would be expected to dampen membrane depolarization due to K^+^ accumulation in the narrow T-tubular space during repetitive muscle stimulation. However another study, by recording chloride currents in the same dissociated mouse flexor digitorum brevis (FDB) muscle fibers before and after disconnection of the T-tubules by osmotic shock, argued for an exclusive expression in the surface membrane (Lueck et al., 2010). A particularly strong point of this latter study is that the authors did not see any significant reduction of ClC-1 currents after detubulation. In this case, the distantly localized sarcolemmal gCl would exert a “remote control” on the T-system guaranteeing an efficient control of the tubular membrane potential (Lueck et al., 2010; Zifarelli and Pusch, 2010). Several arguments supported this view. First, an exclusive sarcolemmal localization of ClC-1 would maintain a very negative chloride equilibrium potential allowing a higher buffering capacity. Second, in the absence of a large gCl in the T-tubules, the resting conductance of the T-tubular membrane would be small, allowing faster and less energy consuming action potential propagation. The more recent study performed using a potentiometric dye to measure the T-tubular potential in a region of a dissociated mouse FDB muscle fiber under two-electrode voltage-clamp, presents evidence for a homogeneous distribution across T-tubules and the outer sarcolemmal membrane (DiFranco et al., 2011). Regardless of their exact localization, ClC-1 channels constitute the main support of the shunting chloride conductance in the skeletal muscle, allowing the fibers to electrically tolerate T-tubular membrane invaginations and the extracellular K^+^ accumulation subsequent to membrane depolarization.

**FIGURE 2:**
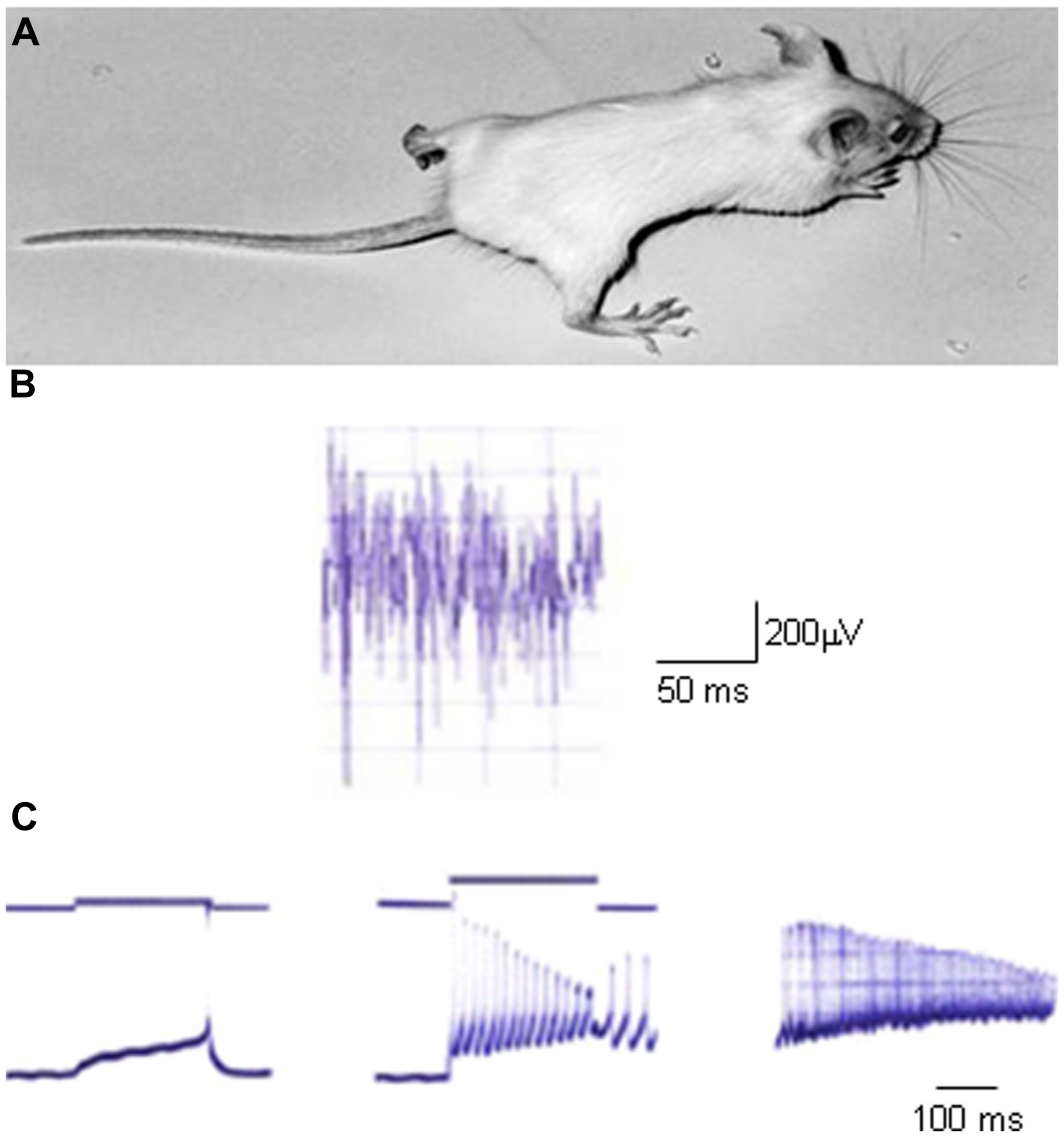
**An animal model of myotonia congenita**. **(A)** Picture of a homozygous adr/adr mouse carrying a loss-of-function mutation in the *clcn1* gene, and showing stiffness in the hindlimb. **(B)** Typical electromyographic activity recorded *in vivo* with needle electrode in the hindlimb musculature of adr/adr mouse. **(C)** Typical electrophysiological recordings of excitability characteristics of adr/adr mouse intercostal muscle fibers with two-microelectrodes current-clamp technique. Left trace shows a single action potential elicited by a threshold current. Note the very long latency between the beginning of current stimulus and action potential onset, due to the reduced sarcolemmal chloride conductance. The middle trace shows the typical myotonic after-discharges, when the fiber fires action potentials after the end of current stimulus. The right trace shows typical myotonic spontaneous activity in absence of stimulus.

The study of myotonia pathomechanisms in muscle fibers from genetically myotonic goats has paved the way for the understanding of the physiological role of ClC-1 channels (Bryant, 1969; Bryant and Morales-Aguilera, 1971; Adrian and Bryant, 1974). Differently from other excitable tissues, the sarcolemma of adult skeletal muscle fibers at rest presents a characteristic high chloride conductance (gCl) that is more than fourfold the potassium conductance (Hodgkin and Horowicz, 1959; Bretag, 1987). Such a large inhibitory Cl^-^ conductance provides the appropriate balance to the excitatory Na^+^ currents to guarantee a constant voltage in any particular region of the fiber. When an action potential is triggered, it is propagated along the surface sarcolemma and deep into the interior of fibers via the T-tubule system. Membrane repolarization initiates after sodium channel fast inactivation and is completed by potassium eﬄux through delayed rectifier potassium channels. In normal skeletal muscle, potassium accumulation in the T-system causes a small, transient after-depolarization at the end of muscle contraction, but the high resting chloride conductance prevents propagation of this depolarization along the sarcolemma (Dutka et al., 2008). Conversely, in myotonic muscle fibers, where the gCl is dramatically reduced by more than 50%, this potassium accumulation can trigger autonomous muscle fiber action potentials, that result in a prolonged electrical activity even after the end of neuronal input (the so-called myotonic after-discharges). This represents the electrophysiological basis for muscle stiffness (**Figure 2**; Adrian and Bryant, 1974; Steinmeyer et al., 1991b).

Recently, a series of elegant studies suggested that during a train of action potentials, the gCl is decreased at the onset of stimulation mainly due to PKC activation, allowing the working muscle to maintain neuromuscular transmission and T-system excitability (Pedersen et al., 2009a,b; Fraser et al., 2011). Nevertheless, after a determined number of action potentials during sustained activity, the gCl is largely increased together with the potassium conductance supported by ATP-sensitive K^+^ channels, most probably in order to terminate muscle activity and prevent metabolic stress that otherwise would damage the muscle fiber (Pedersen et al., 2009a). Interestingly, the rising phase of sarcolemma conductance during the action potentials train was not observed in the slow-twitch soleus muscle fibers, which is consistent with the elevated metabolic capacity of slow-twitch fibers that are rather involved in prolonged contractile activity (Pedersen et al., 2009b). The molecular mechanism responsible for the increased gCl is not well defined. It is likely that the consumption of ATP during prolonged activity may contribute to such an effect, as suggested also by the acceleration of this effect in glucose-free conditions (Pedersen et al., 2009b). The reduction of ATP might increase the gCl by completely removing the ATP- and/or phosphorylation-dependent inhibition of ClC-1 channels. Altogether these data suggest a pivotal role for ClC-1 channels for the modulation of muscular fatigue. Accordingly, the depression of muscle excitability provoked by intense exercise can be antagonized by inhibition of ClC-1 channels through intracellular acidification, addition of lactate, direct pharmacological block by 9-anthracen carboxylic acid (9-AC), or by removal of external Cl^-^ (Nielsen et al., 2001; Pedersen et al., 2004, 2005; de Paoli et al., 2010). The more recent study confirms a biphasic relationship between the gCl and contractile endurance in isolated rat muscles (de Paoli et al., 2013). On one side, the reduction of gCl increases muscle excitability but, on the other side, reduces the capacity to maintain K^+^ homeostasis and membrane potential during contractions.

### Muscle Plasticity

The ClC-1 channels contribute to determine skeletal muscle phenotype and to muscle plasticity. At birth, the sarcolemma gCl is very low, while it increases during the first weeks of postnatal development under the control of nerve input (Conte Camerino et al., 1989). The change in gCl is linearly correlated with changes in ClC-1 channel expression (Steinmeyer et al., 1991a). In adults, the resting gCl is about 10-fold greater than the gK in the fast-twitch muscles, while the slow-twitch muscle phenotype is characterized by a ∼50% lower gCl (Bretag, 1987). This difference is due to a reduced ClC-1 channel expression and an increased basal PKC-dependent phosphorylation of the channel in the slow-twitch muscle (Pierno et al., 2007). As anticipated above, such a phenotypic difference is of critical relevance for the specific modulation of fiber excitability and the consequent contractile function. Several evidences also indicate that the gCl contributes to the determination of muscle phenotype. Indeed, the pharmacological block of ClC-1 channels during the postnatal development forces the fast-twitch muscle to acquire a slow phenotype (De Luca et al., 1990a). Also in adult animals, the block of ClC-1 channels either through natural mutations or pharmacological block favors the transition of fast-twitch muscles toward a less fast phenotype (Salviati et al., 1986; Goblet and Whalen, 1995). Conversely, during the unloading condition, such as that encountered in microgravity condition or in the hindlimb-unloaded rodent model, the slow-to-fast phenotype shift of the postural slow-twitch soleus muscle is preceded by an increase of the gCl (Pierno et al., 2002). Such an increase stems from a reduced activity of the PKC likely due to changes in nerve input (Pierno et al., 2007). This indicates that the increase of gCl constitutes an early event during the adaptation of postural muscle to unloading, which may be necessary to complete the phenotype transition.

### Aging

In the advanced age (>24 months for rats), the gCl can be dramatically reduced in the fast-twitch muscles (De Luca et al., 1990b, 1994a; De Luca and Conte Camerino, 1992). This reduction is attributable to a decrease of ClC-1 channel expression and an increased PKC-dependent phosphorylation of expressed ClC-1 channels (De Luca et al., 1997a; Pierno et al., 1999). The later effect may be related to the aging-related increase of calcium concentration in the muscle fiber (Fraysse et al., 2006). Chronic treatment of aged rats with the growth hormone was able to restore the gCl in fast-twitch muscles, likely through the induction of IGF-1 (De Luca et al., 1994b). In turn, IGF-1 was shown to increase the gCl in aged muscles, at least partially through the activation of an okadaic acid-dependent phosphatase able to de-phosphorylate the channel (De Luca et al., 1997a). All the effects of aging and GH were remarkably fiber type dependent, being less pronounced in the slow-twitch soleus muscle (Fraysse et al., 2006). Interestingly, chronic treatments of aged rats with the amino acid taurine or an olive oil-derived mixture, both with known antioxidant activity, were able to partially counteract the age-related decline of gCl, suggesting that the oxidative challenge during the aging process may be involved in the reduction of the gCl (Pierno et al., 1998, 2014). Based on the role of gCl in the adult fast-twitch muscle, a reduction of gCl in the aged muscle would be expected to increase excitability and force endurance. Nevertheless, the alteration of the entire electrical machinery of sarcolemma, including sodium, potassium, and calcium channels, actually impairs excitability and E-C coupling in the aged muscle (De Luca et al., 1990b, 1992; Tricarico and Camerino, 1994; Delbono et al., 1995; Renganathan et al., 1997; Desaphy et al., 1998).

## Genetic Diseases Associated to ClC-1

### Myotonia Congenita

Mutations in the *CLCN1* gene, leading to partial or complete loss of ClC-1 channel function, are responsible for the pathogenesis of MC (Koch et al., 1992; George et al., 1993; Burge and Hanna, 2012). This is the most common skeletal muscle hereditary channelopathy in humans, characterized by muscle stiffness after a voluntary movement, that is worse after rest, and improves with repeated activity according to the so-called warm-up phenomenon. Inheritance can be dominant (Thomsen’s disease) or recessive (Becker’s disease), with a more severe phenotype in the latter form (Colding-Jørgensen, 2005; Fialho et al., 2007; Raja Rayan and Hanna, 2010). Patients with recessive MC may also have transient weakness at the onset of voluntary contraction, that may lead to falls. Different degrees of myotonic symptoms can be reported among family members with identical ClC-1 mutations and among different families (Colding-Jørgensen, 2005). Chloride channel myotonia also occurs in animals, including goat, mouse, dog, cat, poney, and water buffalo (**Figure 2**; Steinmeyer et al., 1991b; Rhodes et al., 1999; Wijnberg et al., 2012; Borges et al., 2013; Gandolfi et al., 2014). The study of myotonia in goats and mice proved to be decisive to the understanding of the pathomechanism, and eventually allowed the cloning of ClC-1 channel. The electrophysiological correlate of clinical myotonia is a burst of autonomous muscle action potentials that lasts a few seconds after the cessation of motor neuron activity and delays relaxation. Given the role played by ClC-1 channels in muscle fibers, the reduced chloride conductance resulting from ClC-1 mutations will predispose the sarcolemma to spontaneous action potential runs or abnormal after-discharges that hamper muscle relaxation after contraction, causing myotonia (Lossin and George, 2008).

So far more than 130 mutations in *CLCN1* have been identified (Lossin and George, 2008; Matthews et al., 2010) over the entire length of the channel, and the analysis of a number of ClC-1 naturally occurring mutations in heterologous expression systems has contributed to the understanding of the molecular structure and function of the channel and of the disease pathogenesis. The *CLCN1* mutations include small deletions, insertions, frame-shifts, stop codons, missense and splice-site mutations (Esteban et al., 1998; Sangiuolo et al., 1998; Brugnoni et al., 1999, 2013; de Diego et al., 1999; Pusch, 2002; Wu et al., 2002; Fialho et al., 2007; Lossin and George, 2008; Modoni et al., 2011; Mazón et al., 2012). Recently, it was demonstrated that also *CLCN1* exon deletions or duplications in patients with a single identified recessive mutation, can cause recessive MC (Raja Rayan et al., 2012). The majority of MC mutations are predicted to reduce channel expression by defective trafficking or to cause various alterations of channel function including shifts of voltage dependence, reduced single channel conductance, altered ion selectivity (**Figure 1B**, **Table 1**; Tsujino et al., 2011; Ulzi et al., 2012; Weinberger et al., 2012; Desaphy et al., 2013). A novel mechanism has been proposed for the G200R and Y261C mutations, which abolished the potentiation of NAD^+^-induced ClC-1 channel inhibition by low pH_i_ (Bennetts et al., 2012).

**Table 1:** **Diseases associated to dysfunction of *CLCN1* gene**.

Disease	Biophysical defect	Protein defect	Protein domain	Reference
Myotonia congenita	Similar to WT	F167L	C	Ulzi et al. (2012)
	Reduced chloride current due to positive shift of voltage-dependence of activation; no dominant-negative effect on WT	G190S	D	Desaphy et al. (2013)
	Reduced chloride current due to positive shift of voltage-dependence of slow gating; dominant-negative effect on WT	I290M	H-I loop	Pusch (2002)
	Reduced chloride current due to inverted voltage dependence	D136G	C	Stölting et al. (2014b)
	Reduced chloride currents due to reduced single channel conductance, inverted voltage dependence and reduced open probabilities of fast and slow gating	C277Y	H	Weinberger et al. (2012)
	Altered halide selectivity and outward rectification at positive potentials	G230E	H	Stölting et al. (2014b)
	Reduced expression and reduced chloride current due to sarcoplasmic reticulum retention and enhanced proteasomal and endosomal degradation	A531V	O	Papponen et al. (2008), Desaphy et al. (2013)
	Abolished potentiation of NAD^+^-induced ClC-1 channel inhibition by low pH_i_	G200R	D	Bennetts et al. (2012)
Epilepsy	not determined	R976X	C-terminus	Chen et al. (2013)
Myotonic distrophy type I and II	Reduced chloride conductance	Aberrant splicing		Charlet-B et al. (2002), Mankodi et al. (2002)
Huntington disease	Reduced ClC-1 chloride currents	Aberrant splicing		Waters et al. (2013)

*For myotonia congenita, only one representative mutation is listed for each type of biophysical alteration. For an exhaustive review of ClC-1 mutations causing myotonia congenita, see Pusch (2002)*.

Some mutations exhibit both a dominant and recessive inheritance pattern, that complicates our understanding of the genotype–phenotype correlation and mode of inheritance. Remarkably, defects in the fast and slow gating modes of the channel have been proposed to explain the molecular mechanisms for dominant and recessive MC forms (Accardi and Pusch, 2000; Simpson et al., 2004; Weinberger et al., 2012). Furthermore, structure-function relationships in the ClC-1 channel provided relevant information to the understanding of the molecular pathogenesis of MC (Skálová et al., 2013). As a general rule, dominant mutations located on one subunit of the dimer may disrupt the slow gate, thereby exerting a dominant-negative effect on the unaffected associated wild-type subunit. As the slow gate involves subunits interaction, and helices at the dimer interface primarily contribute to cooperative gating (Duffield et al., 2003), this mechanistic hypothesis easily fits to dominant mutations residing on the boundary region between two monomers (Fialho et al., 2007). The homology model of hClC-1 based on the crystallographic structure of CmCLC allowed to identify residues constituting the dimer interface and the ion conducting pore, and indeed to validate experimental data and mechanistic hypotheses (Feng et al., 2010; Skálová et al., 2013). Indeed, most dominant MC mutations proven to cause a dominant-negative effect when co-expressed with WT subunits effectively reside at the boundary region between two monomers or in its proximity. Conversely, mutations occurring in the channel pore show no dominant-negative effect and can cause the disease by directly affecting the channel activity or the local structure of one subunit, or induce misfolding of the mutant subunit (Skálová et al., 2013). On the other hand, recessive Becker mutations present in both alleles may limit expression or affect the fast gate of individual subunits, thereby causing different degrees of haploinsufficiency. Variation in symptoms amongst MC patients cannot be fully explained by the different effect of ClC-1 mutations, since relatives carrying identical ClC-1 mutations can exhibit markedly different phenotypes (Colding-Jørgensen, 2005). In addition, a number of ClC-1 mutations found in myotonic carriers show little or no evidence of defective channel function in heterologous expression systems (Pusch, 2002; Simpson et al., 2004; Desaphy et al., 2013; Lucchiari et al., 2013). One possibility is that these variants might actually modify the phenotype of a co-existing MC mutation without being frankly pathogenic. Alternatively, these mutations might impair channel modulation by signaling pathways or accessory proteins in the muscle context. In addition, difference in allelic expression of *CLCN1* may influence the clinical phenotype (Duno et al., 2004). Last but not least, other genes may be involved in the phenotype variability, acting as disease modifiers, although such factors have not been identified so far (Colding-Jørgensen, 2005). Myotonic symptoms are known to worsen during pregnancy and with use of diuretics (Bretag et al., 1980; Colding-Jørgensen, 2005; Basu et al., 2009), both conditions being associated with increased risk of hypomagnesemia and hypocalcemia (Raman et al., 1991; Skov et al., 2013). Indeed, the elevation of [Mg^2+^]_o_ or [Ca^2+^]_o_, within their physiological ranges, dampened 9-AC-induced myotonia in isolated rat and human muscle fibers (Skov et al., 2013, 2015).

### Myotonic Dystrophy

Conversely to MC, myotonic dystrophy is an autosomal dominant multisystemic disease due to expansion of nucleotide repeats in the DMPK (DM1 or Steinert disease) or ZNF9 (DM2 or proximal myotonic myopathy or PROMM) genes (Meola, 2013; Meola and Cardani, 2014). It is the commonest form of muscular dystrophy in adults. In both forms, the nucleotide expansion is transcribed in RNAs that accumulate in the cell nucleus and constitute the so-called foci, which affects the transcription and splicing of various genes. In particular, DMPK- and ZNF9-induced alteration of *CLCN1* transcription have been demonstrated in several mouse models of DM1 and DM2, and is likely responsible for the observed myotonia (Charlet-B et al., 2002; Mankodi et al., 2002; Chen et al., 2007; Lueck et al., 2007; Wheeler et al., 2007; Hao et al., 2008).

In addition, ClC-1 mutations have been found in diagnosed DM2 patients, which worsen the disease. For instance, the ClC-1 mutation R894X was shown to enhance myotonia and pain in DM2 patients (Ursu et al., 2012). In another DM2 patient, the F167L ClC-1 mutation might explain the unusual early onset of the disease (Cardani et al., 2012). Thus, it has been hypothesized that DM2 patients with co-segregating *CLCN1* mutation have an increased likelihood to be referred for molecular diagnostic testing owing to the exacerbation of the clinical phenotype (Suominen et al., 2008).

The gCl is also an index of the dystrophic condition of dystrophin-deficient muscles in the mdx mouse, a model of Duchenne muscular dystrophy (De Luca et al., 1995). Indeed, the diaphragm and extensor digitorum longus (EDL) muscles from mdx mice show a decrease of gCl and an increase of gK. These changes were very dramatic in diaphragm fibers suggesting that the impairment of gCl in the degenerating diaphragm could account for some of the symptoms of the human disease (De Luca et al., 1997b).

### Epilepsy and other Neurological Disorders

Epileptic phenotypes resulting from defects in a number of ion channel types have been recently classified as “*channelepsies*” (D’Adamo et al., 2013). Evidence for a role of ClC-1 channels in the pathogenesis of “*Cl^-^ -channelepilepsy”* is derived from large-scale exome analysis of ion channel variants and expression studies (Chen et al., 2013). Indeed, the *CLCN1* gene has been recently proposed as a candidate gene for epilepsy. The analysis of a cohort of patients suffering from idiopathic epilepsy showed that the occurrence of single nucleotide polymorphisms in *CLCN1* was threefold higher in affected patients compared to controls. Moreover, a novel ClC-1 premature stop codon (R976X), resulting in truncation of the distal C-terminus of the protein, was identified by exomic sequencing in one patient with generalized pharmacoresistant epilepsy and mild myotonic features (Chen et al., 2013). The same study also showed unexpected ClC-1 mRNA transcripts and ClC-1 protein bands in several human brain areas, including hippocampus, cerebellar Purkinje cell layer, brainstem nuclei, frontal neocortex and thalamic nuclei.

This novel localization of ClC-1 might open new perspectives regarding the role of CLC channels in the central nervous system. First, if ClC-1 is expressed in human brain, then mutations that reduce chloride conductance could contribute to enhanced network excitability and increased susceptibility to seizures or other neurologic phenotypes. A second and intriguing observation is the presence of ClC-1 in subcortical structures, such as the basal ganglia, subthalamus, and cerebellar Purkinje cell layer, which raises the possibility that dysfunction of brain ClC-1 might contribute, in some individuals carrying ClC-1mutations, to the dystonia phenotype which has previously been regarded as being exclusively of muscular origin.

However, patients with MC and related animal models do not experience central neurological symptoms, and to date only one single case of ClC-1 mutation associated to seizures has been reported. An hypothesis to explain the low occurrence of ClC-1-associated epilepsy phenotype considers a compensatory mechanism possibly involving ClC-2, given the extensive overlapping in the regional brain expression of both channels, and the possible formation of heteromeric channels (Chen et al., 2013; Stölting et al., 2014a).

The association between hereditary myotonic disorders and brain diseases has been rarely reported in the literature. The first case of association between familial Thomsen myotonia and epilepsy has been presented very recently, with the description of one individual belonging to a two-generation family suffering from ClC-1-linked myotonia, who experienced epileptic seizures due to limbic encephalitis with antibodies against glutamate decarboxylase (Licchetta et al., 2014). Another recent paper have reported in a boy the co-existence of a ClC-1 mutation causing recessive MC and of a PMP22 mutation causing dominant Charcot-Marie-Tooth disease type 1A (Ardissone et al., 2013). These few “double trouble” cases do not allow to define whether the concomitant genetic condition may influence the severity and progression of the diseases.

A reduction of ClC-1 channel activity has recently been described in skeletal muscles of a mouse model of Huntington disease (HD; Waters et al., 2013). As in myotonic dystrophy, HD is due to the expansion of a trinucleotide repeat in the HD gene, and improper *clcn1* mRNA splicing was shown to compromise ClC-1 channel expression and function. The muscle fibers of HD mouse were hyper-excitable, with a reduction of threshold to trigger action potentials, an increased number of fired action potentials, and the occurrence of spontaneous action potentials, eventually causing involuntary and prolonged contractions. It was thus proposed that ClC-1 impairment likely contribute to the chorea, stiffness, and dystonia that characterize HD (Waters et al., 2013).

## Pharmacology of ClC-1

Much of the information regarding ClC-1 channel pharmacology has been obtained by studying skeletal muscle chloride conductance and heterologously expressed ClC-1 channels, leading to the identification of a series of poorly selective blockers and a few activators (**Table 2**).

**Table 2:** **Drugs acting on skeletal muscle gCl and ClC-1**.

Drugs	Effect on skeletal muscle	Mechanism of action	Reference
Acetazolamide	Improves myotonic symptoms in affected patients	Increases ClC-1 open probability likely via a pH_i_-dependent effect	Eguchi et al. (2006), Matthews et al. (2010)
Insulin-like growth factor-1	Increases sarcolemma gCl	Increases ClC-1 dephosphorylation	De Luca et al. (1998)
Taurine	Increases sarcolemma gCl	Not determined	De Luca et al. (1998)
Statins	Reduce sarcolemma gCl	Increase PKC-dependent ClC-1 phosphorylation	Pierno et al. (2006, 2009)
R(+)-CPP	Increases sarcolemma gCl	Not determined, since it blocks heterologously expressed ClC-1	De Luca et al. (1992), Pusch et al. (2000)
9-AC	Reduces sarcolemma gCl and induces myotonic symptoms	Blocks heterologously espressed ClC-1; binds to the pore from intracellular side	Estévez et al. (2003), Desaphy et al. (2014)
Fenofibrate, clofibrate and their metabolites (CPP and CPA)	Reduce sarcolemma gCl and induce myotonic symptoms	Block heterologously espressed ClC-1; CPP and CPA bind to the pore from intracellular side	Liantonio et al. (2002), Estévez et al. (2003), Pierno et al. (2006, 2009)
Niflumic acid	Reduces sarcolemma gCl	Blocks heterologously espressed ClC-1; binds to the pore from intracellular side and increases PKC-dependent phosphorylation	Liantonio et al. (2007)

*9-AC, 9-anthracen carboxylic acid; CPP, 2-(p-chlorophenoxy) propionic acid; CPA, p-chlorophenoxyacetic acid*.

The interest in ClC-1 pharmacology stems primarily from the lack of a specific pharmacological treatment for MC patients. Today, mexiletine, a sodium channel blocker, represents the first line therapy for the myotonic syndromes, irrespective of the culprit gene. This drug can be effective in reducing stiffness and transient weakness in MC and myotonic dystrophy, but its use is limited by country availability, side effects and suboptimal or negative response in some patients (Logigian et al., 2010; Statland et al., 2012; Lo Monaco et al., 2015). The ideal drug to treat ClC-1 channelopathy should be one able to increase chloride currents. However, this goal is far from being achieved. Two carbonic anhydrase inhibitors, acetazolamide and diclorfenamide, have been used empirically for their beneficial action on a variety of neurological disorders including myotonia with variable results (Griggs et al., 1978; D’Adamo et al., 1999, 2012, 2014, 2015; Imbrici et al., 2008; Matthews et al., 2010; Markhorst et al., 2014). One possible mechanism accounting for membrane electrical stabilization by acetazolamide consists in the opening of calcium-activated potassium channels (Tricarico et al., 2000). More recently, it was proposed that this drug is also able to shift ClC-1 open probability toward more negative voltage, probably through change in pH_i_, resulting in an increased chloride current and consequently accounting for an antimyotonic effect (Eguchi et al., 2006; Desaphy et al., 2013). It is, however, possible that the significant percentage of non responders stems from a reduced sensitivity of some mutant channels to the drug (Desaphy et al., 2013).

In the past, two electrode voltage clamp recordings from muscle fibers revealed that the R(+)-isomer of 2-(p-chlorophenoxy) propionic acid (CPP) was able to increase the skeletal muscle chloride conductance at concentrations as low as 1–5 μM, making it a promising compound in therapy (Conte-Camerino et al., 1988; De Luca et al., 1992). However, the opener activity of R-CPP was not observed when applied to heterologously expressed ClC-1 channels (Aromataris et al., 1999; Pusch et al., 2000), suggesting an indirect action through a muscle-specific component in the native system. Another attempt to enhance chloride currents has been to indirectly increase ClC-1 channel expression or function by stimulating intracellular biochemical pathways. For instance, muscle chloride conductance increases *in vivo* and *in vitro* after application of IGF-1 or taurine (De Luca et al., 1996, 1998). PKC inhibitors, such as staurosporine or chelerythrine, has been shown to modulate ClC-1 channel (Rosenbohm et al., 1999) and increase gCl (De Luca et al., 1994a; Pierno et al., 2003), as above described. Some of them already used in therapy to treat cancer (i.e., enzastaurin) might be considered for ameliorating ClC-1 channel loss of function. Only recently, lubiprostone, a bicyclic fatty acid derivative, approved for the treatment of idiopathic chronic constipation, has been shown to selectively activate ClC-2 channels without activation of prostaglandin receptors (Lacy and Levy, 2007). Further studies would be necessary to verify whether such compound may serve as lead compound for developing future ClC-1 openers.

Although they lack a direct therapeutic interest, compounds able to block gCl have proved useful pharmacological tools to develop animal models of myotonia or to investigate hClC-1 structure-function relationship. For instance, through the reduction of gCl, 9-AC has been largely used to reproduce myotonia in isolated muscles or *in vivo* (Bryant and Morales-Aguilera, 1971; Skov et al., 2013; Desaphy et al., 2014). Also, clofibrate induces a myotonic-like state in rodents blocking gCl in a dose-dependent anner (De Luca et al., 1992). A considerable number of clofibric acid derivatives have been developed and tested in native skeletal muscle fibers and in heterologously expressed hClC-1 channels in order to find the structural determinants for a selective channel block. The most active compound resulted to be the S(-) isomer of CPP, capable of inhibiting ClC-0 and ClC-1 with higher potency than ClC-2 (Pusch et al., 2000; Liantonio et al., 2002). This compound induces a voltage-dependent block, reducing currents at negative voltages, thus indicating a higher affinity toward the closed state of the channel (Aromataris et al., 1999; Pusch et al., 2001). The p-chlorophenoxyacetic acid (CPA), as 9-AC, exerts its blocking activity by binding to an hydrophobic pocket within the channel pore (Accardi and Pusch, 2003; Estévez et al., 2003). Like clofibrate, fenofibrate directly blocks heterologous-expressed hClC-1 channels, and determined a reduction of gCl in rat skeletal muscles after *in vivo* treatment (Pierno et al., 2006, 2009).

Also the widely-used cholesterol lowering medications, statins, have been found to reduce the gCl in rat muscles, which may contribute to the common side effects of the drugs on muscles. The effects of statins on the gCl is, however, indirect, involving the PKC-dependent phosphorylation of ClC-1 channels or a reduction of ClC-1 expression (Pierno et al., 2006, 2009).

Interestingly, niflumic acid, a non steroidal anti-inflammatory drug, has been found to decrease muscle gCl by blocking ClC-1 channels both through direct binding and through Ca^2+^-dependent phosphorylation by PKC (Liantonio et al., 2007).

## Challenges

A wealth of information about ClC-1 channels have been acquired during the 25 years since the cloning of the first member of the CLC family. However, several questions still await an exhaustive answer and represent major challenges to next research. The molecular identity and mechanism of the slow gating is still debated and the functions of the two CBS domains is still under investigation. Little is known about ClC-1 modulation by intracellular and extracellular factors and, despite the fact that the role of gCl in skeletal muscle plasticity has been assessed, the underlying molecular mechanisms are not completely understood. Future investigation will be committed to understand the possible role of ClC-1 in the brain and its involvement in the etiopathogenesis of epilepsy, as well as to face the still unclear aspects of MC, such as the lack of a precise genetic diagnosis for several patients, the exact molecular mechanisms for dominant and recessive inheritance, the mechanisms underlying myotonic symptoms in patients carrying apparently silent mutations. One major challenge regards the possibility to guarantee an appropriate and personalized therapy to MC patients through the identification of specific pharmacological strategies able to counteract the reduction in chloride conductance that causes MC (Trivedi et al., 2014). It is expected that the experimental data already available on ClC-1 blockers, through molecular dynamics simulations and functional studies, may offer a solid pharmacophore model for developing selective ClC-1 openers.

## Conflict of Interest Statement

The authors declare that the research was conducted in the absence of any commercial or financial relationships that could be construed as a potential conflict of interest.
